# Effect of Particle Size and Shape on Wall Slip of Highly Filled Powder Feedstocks for Material Extrusion and Powder Injection Molding

**DOI:** 10.1089/3dp.2021.0157

**Published:** 2023-04-12

**Authors:** Daniel Sanetrnik, Berenika Hausnerova, Martin Novak, Bhimasena Nagaraj Mukund

**Affiliations:** ^1^ Centre of Polymer Systems, University Institute, Tomas Bata University in Zlin, Zlin, Czech Republic.; ^2^ Department of Production Engineering, Faculty of Technology, Tomas Bata University in Zlin, Zlin, Czech Republic.; ^3^ Indo MIM Pvt. Ltd., Bangalore, India.

**Keywords:** material extrusion, powder injection molding, highly filled feedstock, wall slip, powder shape, particle size, die geometry

## Abstract

A necessity to distinguish between the influence of powder shape and size (particle size distribution) is especially demanding for highly filled metal powder feedstocks employed in additive manufacturing and powder injection molding. As their processability is evaluated through rheological behavior, the study focuses on the effect of powder size/shape on a wall slip, which is a typical phenomenon determining flow performance of these materials. Water and gas atomized 17-4PH stainless steel powders with *D*_50_ of about 3 and 20 μm are admixed into a binder containing low-density polyethylene, ethylene vinyl acetate, and paraffin wax. Mooney analysis to intercept the slip velocity of 55 vol. % filled compounds reveals that wall slip effect appears to vary significantly with size and shape of metal powders—round shaped and large particles are the most prone to the wall slip. However, the evaluation is affected by the type of the flow streams resulting from the geometry of the dies—conical dies reduce the slip up to 60% in case of fine and round particles.

## Introduction

Although additive manufacturing is merging with powder injection molding (PIM) in the field of production of highly precise metal items and gaining relevant attraction due to a combination of processability of plastics and mechanical, electric or magnetic properties of metals,^1^ there are still fundamental issues not answered satisfactorily. An entire manufacturing process consists of four steps (mixing, material extrusion [MEX] additive manufacturing or PIM, debinding, and sintering) of an equal importance, which (if performed correctly) lead to the final metallic items of tight tolerances and high quality.^2^ MEX and PIM have common three out of four steps; in case of MEX, there is an additional demand on sufficiently flexible filaments,^3–5^ which however might be eliminated with extrusion designs based on granulate systems.^6,7^

Owing to the complexity of a multistep processing as MEX and PIM technology the current research is focused on the methods capable to intercept and reduce identified issues and defects prior sintering, that is, when the material can still be regranulated and reused.^8–10^ One of the serious common complications is a wall slip during shearing.^11–13^

Currently, the extrusion flow simulations are based on rheological models without considering slip, which however alters the flow data of highly filled compounds substantially. Full understanding of a wall slippage together with reliable flow models^14–16^ and accounting for a pressure-dependent viscosity^17^ would lead to precise simulations of a forming process (flow patterns during extrusion or injection molding as a function of pressure, temperature, and velocity) and avoiding classical time-consuming and costly trial-and-error experiments to set up processing conditions of the forming steps.

As found out by LeBlanc *et al.*^18^ and Saidy *et al.,*^19^ technological limitations of additive manufacturing arise mostly from unstable behavior of polymer binders. Wall slip has been recently linked to the extrudate distortions and overall extrusion defects observed in the capillary flow of highly viscous polymer systems, and it may limit adhesive strength of deposited filaments.^20^ The presence of domains deformed along the angular direction of the extrudate may also be attributed to slip.^21^ Vadodaria *et al.*^22^ reported heterogeneous flow profiles across the measurement gap due to the discontinuous shear gradient across the measurement gap as a consequence of wall slip during measurement of cellulose systems widely used in 3D printing.

Wall slip effect on the extrudability and windability of filaments was recently confirmed by Hasib *et al.*^23^ for feedstocks based on polylactic acid and Ni–Cu powders. Furthermore, the relation between yield stress and shear stress at the wall affected by wall slip was investigated in some studies^24–26^; the wall shear stress higher than the yield stress potentially leads to the phase separation and inhomogeneous print quality. In contrast, wall slip phenomenon might prevent clogging of the nozzle,^27^ but binder rich areas at filament walls might not ensure quality of sintered parts.

In general, the wall slip theory of highly filled polymers^28^ assumes that upon shearing a narrow polymer layer of low viscosity with a typical thickness of 0.1–1 μm is created near the wall, and solid particles migrate to the middle of the flow channel. This means that a homogenous distribution of a powder within a polymer binder is disrupted,^29–31^ and compounds show a phase separation that has a detrimental effect on final sintered structures. Delime and Moan^32^ assumed that the migration of solid particles is initiated by a failure of Brownian movement near walls, which is supported by shear rate gradients, which promote particle collisions.^30,32^ This theory was experimentally confirmed by Lam *et al.*^33^ for the particles ranging from 20 to100 μm.

The magnitude of the wall slip depends significantly on a roughness and chemical nature of a processing tool used. For example, stainless steel was found to be more prone to a wall slip than aluminum,^34^ and the rough surfaces, where solid particles can move into a groove, may suppress the formation of a low molecular layer on channel walls and reduce slip.^35^

During testing of metal powders (316L, 17–4PH) in a catalytic binder system as well as ZrO_2_ feedstocks the same trend was obtained with the viscosity strongly affected by the surface roughness (smooth surfaces led to the lower viscosity and higher wall slip velocity).^36^ These results corresponded to those of pure polymers^34^ as well as suspensions containing polymer matrix poly(butadiene–acrylonitrile–acrylic acid) filled with glass spheres (particles mean size 35 and 85 μm)^37^ and poly(methyl methacrylate) solid spheres (121 μm) in hydroxyl-terminated polybutadiene.^38^ So far, smooth flow channel walls and small channels were found to be the most significant factors causing wall slip.^30,39^

The metal powder compounds for MEX and PIM contain particles of broad particle size distributions (typically from 0.1 to 20 μm) and various shapes—small particles ensure faster sintering but slow down debinding, spherical shape improves flow but irregular enhances a component strength before sintering, wide size distribution lowers sintering shrinkage but slows down debinding, and promotes quality issues and inhomogeneities in a structure.^40,41^

In this article, the materials investigated were selected based on our previous studies of the effect of shape and size of steel powders on the overall processing performance of highly filled compounds, where it was found that coarser (mean size of 11 and 20 μm) gas (GA) atomized particles are required to lower mixing torque, obtain higher critical solid loading and lower viscosity due to the lower friction between particles. However, in case of water (WA) atomized powders, fine powders (mean size of 3 and 8 μm) show a better performance.^42^ Furthermore, it was found out that only a slight variation in particle size distribution fractions significantly influences the quality of the green components and dimensional tolerances of the complex-shaped components.^43^

The effect of powder shape and size is addressed in this study through a thorough analysis of the wall slip with the help of Mooney^44^ method of determination of slip velocity. To our best knowledge, this important aspect of MEX has not been reported heretofore. Successful MEX requires tailored particle size distribution at multiple scales—particle sizes capable of retaining compound cohesion to enable flow as well as provide enough yield stress to the mixture, shape stability, and green strength. Size distribution is also a contributing factor to liquid phase migration under extrusion pressure, leading to flow-induced heterogeneities in the printed structure.^25^ If one considers that the thickness of the polymer layer created near the channel wall during slippage is proportional to the square root of the wall slip velocity,^45,46^ it may form in the critical places of the proceeded specimen, and thus cause the collapse of its surface or even bulk during sintering.^47^

Moreover, as demonstrated in our recent article,^48^ the application of flat or conical dies might affect the rheological data obtained for highly concentrated particulate systems. Thus, the possible influence of an entrance angle of capillary dies on the evaluation of wall slip will be considered in this study as well.

## Experimental

### Materials

GA and WA atomized stainless steel powders 17–4PH with *D*_50_ of about 3 and 20 μm were provided by IndoMIM Pvt. Ltd., India. The particle size distribution of powders (Table 1) was measured using laser diffraction method (Malvern Mastersizer 3000).

**Table 1.** Particle Size Distribution of Powders

Powder	D_10_ (μm)	D_50_ (μm)	D_90_ (μm)
3GA	1.7	2.9	4.8
3WA	1.5	3.0	6.1
20GA	6.7	21.9	49.2
20WA	7.2	23.8	57.0

GA, gas; WA, water.

**FIG. 1.**
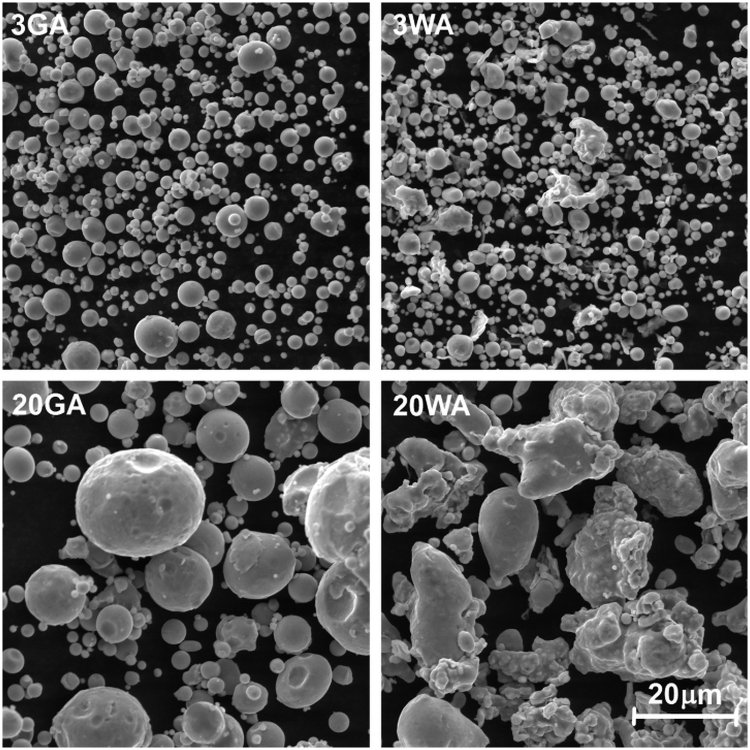
SEM of GA and WA atomized 17-4PH stainless steel powders. GA, gas; SEM, scanning electron microscope; WA, water.

The morphology of powders was observed with a scanning electron microscope (VEGA II, TESCAN) as shown Figure 1.

### Feedstock preparation

Feedstocks were prepared using low-density polyethylene, ethylene vinyl acetate, and paraffin wax-based binder system developed by Hausnerova *et al.*^49^ (Table 2). The values of melting temperatures and densities were taken from the datasheets of the particular suppliers. Powder and binder were compounded using Z-blade laboratory mixer MZ05 (Winkworth) at 140°C for 20 min and a blade speed of 25–30 RPM. An optimum solid loading of 55 vol. % powder was set as 4 vol. % lower than the critical solid loading value of 20WA (lowest from the materials investigated, Table 3). The critical solid loading of powders was measured using the mixing torque method by plastometer Brabender W50; the detailed description of the method can be found in our previous study.^42^

**Table 2.** Relevant Physical Properties of Binder Polymers

Polymer	Content (wt. %)	Density (g/cm^3^)	Melt temperature (°C)
LDPE^a^	53	0.914	102
EVA^b^	26	0.948	67
PW^c^	21	0.900	50

^a^ ExxonMobil™ LDPE LD 650 Low Density Polyethylene Resin.

^b^ ExxonMobil Escorene™ Ultra UL 40028CC Ethylene Vinyl Acetate Copolymer Resin.

^c^ FAGRON Paraffinum Solidum.

**Table 3.** Critical Solid Loading (Vol. %)

3 μm		20 μm	
Gas	Water	Gas	Water
61	62	65	59

### Wall slip analysis

**FIG. 2.**
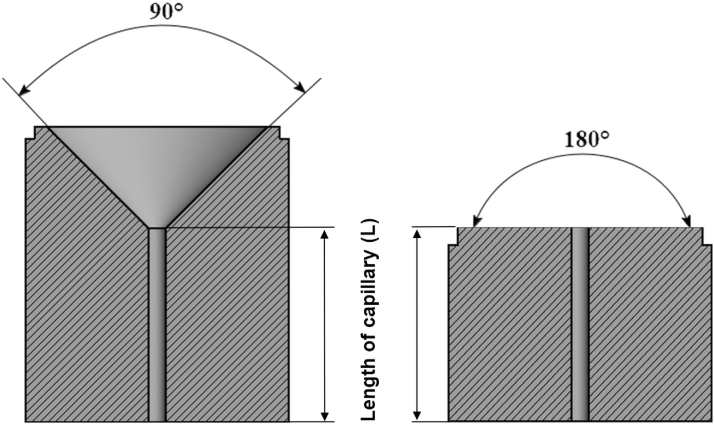
Schematic representation of conical (90°) and flat (180°) testing capillaries.

Wall slip was evaluated using a capillary rheometer (Göettfert 50; Göttfert Werkstoff-Prüfmaschinen GmbH, Buchen, Germany). Mooney rheological analysis was performed on the conical (90°) and flat (180°) dies (Fig. 2) with length to diameter (*L/D*) ratios of 5/0.5, 7/0.7, and 10/1 at 150°C. The diameter of the dies was kept between 0.5 and 1 mm, because the larger capillary diameter than 1 mm might result in an unstable pressure,^48^ and smaller diameters than 0.5 caused blocking of the dies. Pressures used were 35, 42, 50, 59, 69, 88, 109, 137, 185, and 240 bars to cover uniformly as wide range of pressures as possible. Mooney method is based on changing the surface-to-volume ratio of the capillary dies, that is, changing length *L* and radius *R* of the dies, whereas their ratio is kept constant. In a circular die an apparent wall shear rate ${\dot{\gamma }}_{a}$and an apparent wall shear stress $\tau_{a}$ are determined from the relations:

(1) $${\dot{\gamma }}_{a}=\frac{4\dot{Q}}{\pi R^{3}};\tau_{a}=\frac{\Delta pR}{2L},$$

where $\dot{Q}$ is the volumetric flow rate and Δp is the measured pressure drop.

Consequently, a true average velocity is given by the difference between an average *v_av_* ( = $\dot{Q}∕\pi R^{2}$) and a slip *v_slip_* velocities:

(2) $$v_{true}=v_{av}-v_{slip}.$$

Multiplying this relation by 4/*R* and using the relation (1) a dependence of a corrected apparent shear rate on a measured apparent shear rate and wall slip velocity is obtained:

(3) $${\dot{\gamma }}_{a,slip-corrected}={\dot{\gamma }}_{a}-\frac{4v_{slip}}{R}.$$

**FIG. 3.**
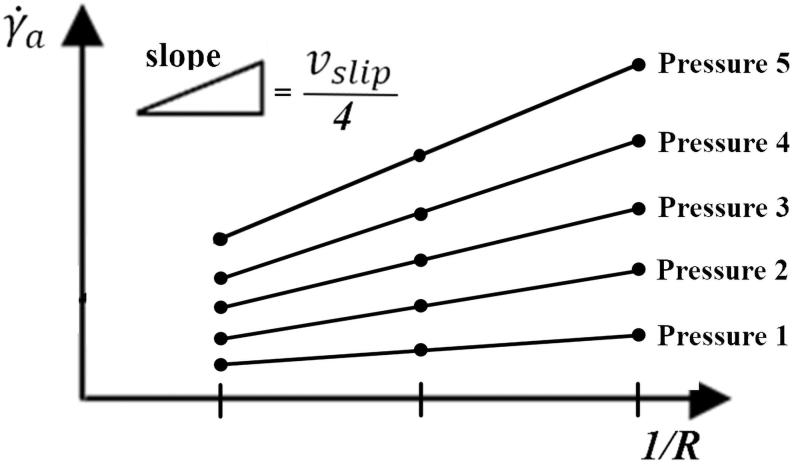
Schematic of a wall slip velocity evaluation using Mooney analysis.

The measurements at constant shear stress (pressure) have to be made with capillaries of different diameters, but the same *L/D* ratio. The obtained apparent shear rate values (${\dot{\gamma }}_{a}$) are plotted into the graph as the function of the reciprocal radius of the capillary die (Fig. 3). The values of each measured pressure are then interpolated by linear regression, which is described by Equation 3. Finally, wall slip velocities are calculated from the slopes (a slope is equal to $4v_{slip}$) of these linear fittings, and expressed as a function of the shear stress in the graph.

## Results and Discussion

**FIG. 4.**
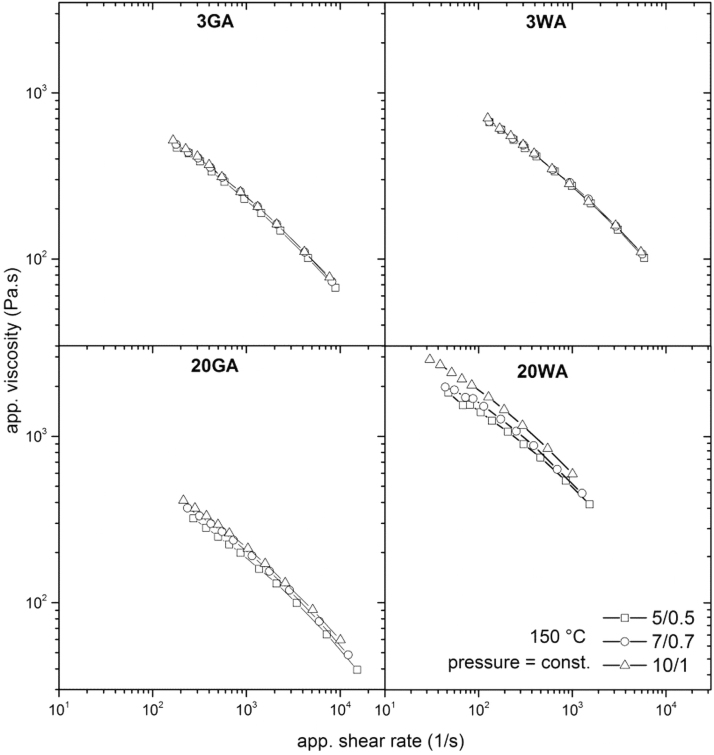
Apparent viscosity as a function of apparent shear rate of 17-4PH feedstocks varying in size and shape obtained with flat capillary dies (entrance angle 180°).

According to the apparent data, all tested feedstocks exhibit a pseudoplastic behavior at 150°C as can be seen in Figure 4 for flat capillary dies. The data obtained for conical capillary dies were similar (Supplementary Fig. S1). Three of the tested materials (3GA, 3WA, and 20GA) show similarly low apparent viscosity values in the range of 80–600 Pa s, which may indicate their slippage at the wall. The apparent viscosity of irregular 20WA compound is about a decade higher than the values of other compounds. This is attributed to the irregular shape of particles, because higher friction of the irregular particles^50^ might overcome the effect of a size in case of coarse particles.^42^ Partly, there is also a possible effect of the overall lowest critical solid loading of this compound (59 vol. %) in comparison with other compounds tested (Table 3).

**FIG. 5.**
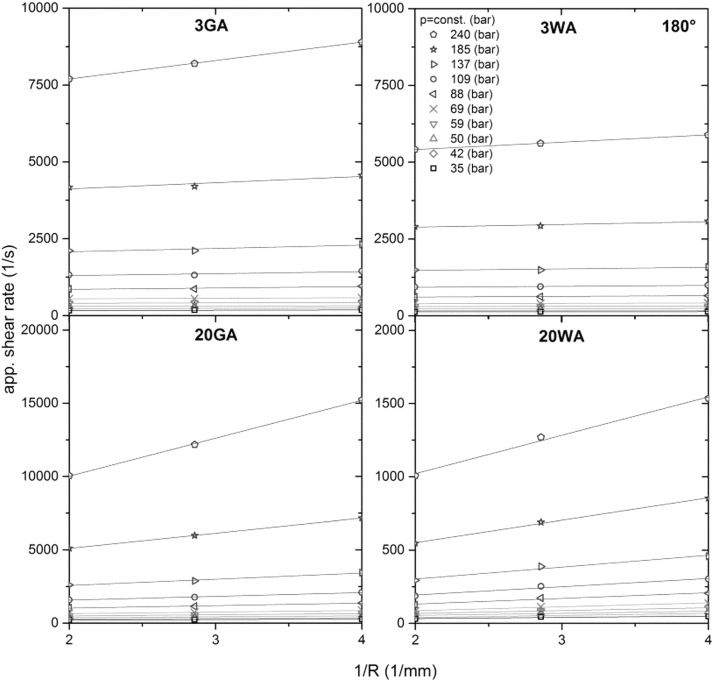
Mooney diagrams of 17-4PH feedstocks varying in size and shape obtained with flat capillary dies (entrance angle 180°).

As can be seen in Figure 5, representing the Mooney plots for the flat capillary dies (for conical capillary dies see Supplementary Fig. S2), an increasing tendency toward the wall slip with increasing shear stress/pressure is obvious from the slopes of the fitting lines of Mooney plots. The linear fitting lines with coefficients of determination higher than 0.995 were obtained.

**FIG. 6.**
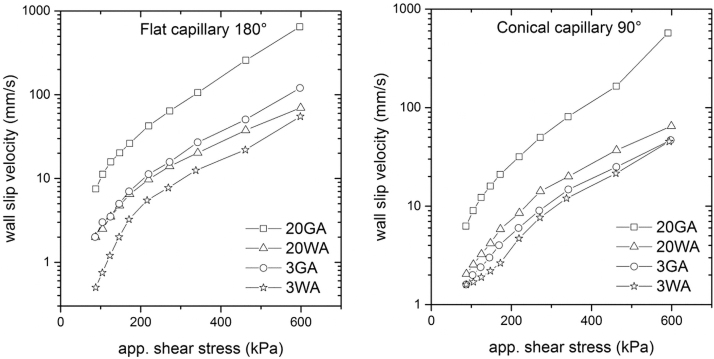
Wall slip velocities of 17-4PH feedstocks affected with powder shape and size obtained at various shear stresses on flat (entrance angle 180°) and conical (entrance angle 90°) dies.

The calculated values of the wall slip velocity (Fig. 6) clearly show the influence of the size and the shape on the wall slip phenomenon, which consequently governs the overall flow performance of the materials during processing. According to our best knowledge there is no report heretofore on the effect of the shape of the metal particles on the wall slip. The feedstocks containing spherical (GA) particles exhibit higher wall slip velocities than irregularly (WA) shaped powders of the comparative sizes, suggesting that spherical particles are more prone to wall slip. It is known that the slip of highly concentrated materials is connected to the separation of powder and binder—particles migrate to the middle of the flow channel due to the shear rate gradients during processing, and the low viscosity polymer layer forms near the die wall.^11,51–53^ In this perspective, the spherical particles seem to be more sensitive to shear rate gradients; as they do not have any preferential orientation dimension (aspect ratio is near 1), they can more easily start to rotate upon shearing as described by Thornagel^54^ and move to the center of the die allowing for the formation of the polymer slip layer. This, as mentioned in Introduction, may lead to an anisotropic shrinkage and other associated defects inside final sintered products.^2,55^ In contrast, WA atomized particles having a flake-like shape tend to orient with the flow front and sustain shearing without losing the uniform distribution within the polymer medium.

If the effect of particle sizes is considered, the data depicted in Figure 6 confirm the findings obtained by Sanetrnik *et al.*^36^ and Chin *et al.,*^56^ where the larger particles were more prone to the wall slip than smaller particles, because fine particles more easily fill the space near the wall, and thus prevent the formation of the polymer layer.

Based on these results, processing of small and irregular particles seems to be more advantageous in terms of a wall slip. Furthermore, it is in accordance with the effect of small particles in reducing powder/binder separation and improving a product homogeneity reported in.^51,57^ At this point it should be pointed out that highly concentrated compounds for PIM and MEX undergo the changes of the particle shape and size during processing as recently reported by Bek *et al.,*^58^ which might be also favorable in reducing a slip.

As can be further noted from Figure 6, the flat dies, which are commonly used in a vast majority of current studies,^28,59^ result in the different wall slip velocities than the values obtained with the conical dies. Liang^60^ and Ardakani *et al.*^61^ tested changes in a pressure drop for different capillary entrance angles during extrusion, and showed that the geometrical arrangement of capillary dies of rheometers may significantly affect rheological data; in their case, the shear rate increased with the capillary entrance angle under the constant pressure. Furthermore, Sanetrnik *et al.*^48^ reported that capillary entrance angles may affect the accuracy of the flow data, and consequently flow simulations carried out to optimize processing. The application of the conical dies was validated as more beneficial for highly filled materials as they do not cause rapid changes in shear rates.

**FIG. 7.**
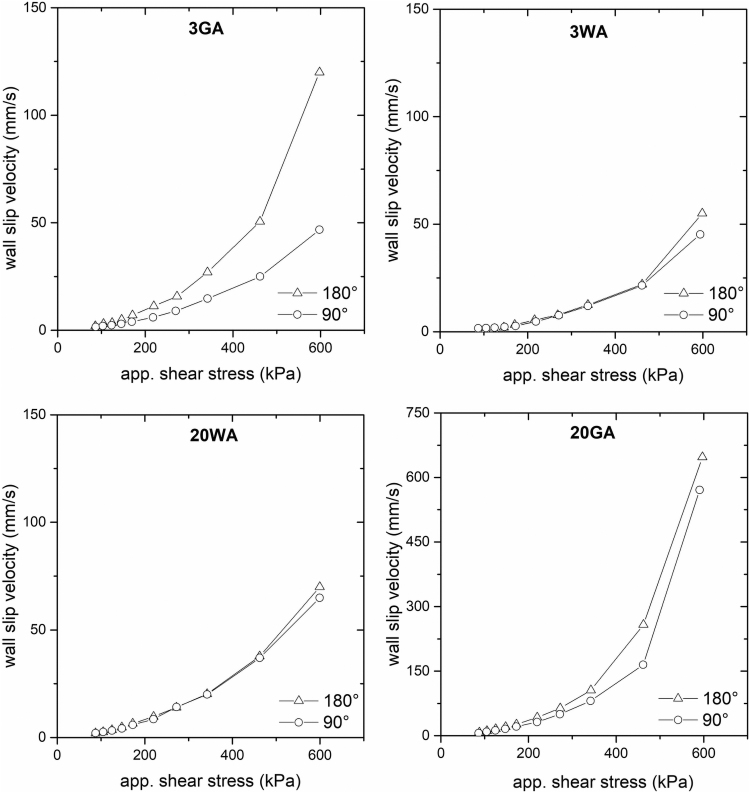
Influence of capillary die entrance angle on wall slip velocity of 17-4PH feedstocks varying in size and shape (note different magnitudes of y-axes).

As can be seen from Figure 7, the wall slip velocities of all tested materials at higher shear stresses, which are usually used in MEX as well PIM, obtained with the conical capillaries are lower than those obtained with the flat dies, whereas at lower shear stresses the differences are rather negligible. Especially, fine and regular particles (3GA) react sensitively to the change of the entrance to the capillary die—at shear stresses about 600 kPa the wall slip velocity was reduced up to 60% with a conical dies. Based on these results and previous research^29–31,62^ it is assumed that highly pronounced slip of small and regular particles measured on flat capillary dies may be attributed to their higher sensitivity to the changes of shear rate gradients at the entrance to the capillary, which are increasing function of entrance angle. This result is also supported with a flow pattern proposed for highly filled compounds by Thornagel.^54^ As regular and fine particles are assumed to be more free to rotate and leave areas with higher shear rate gradients, they are more sensitive to the shear rate changes during flow in a capillary as well.

According to these findings, utilizing lower print/injection speeds, conical dies, loadings closer to a critical value, and generally ensuring more viscous melts may help to reduce wall slip, and consequently powder/binder separation resulting in defects on sintered parts.

## Conclusion

Highly filled (55 vol. %) MEX and PIM compounds based on metal powders varying in shape and size are sensitive to wall slip as a crucial processability parameter whose neglecting results in an inaccurate rheological data. The effect of the shape of the particles not investigated heretofore was found to be more pronounced than the effect of the size. Spherical (GA atomized) and large (20 μm) particles appear to be more sensitive to wall slip than irregular (WA atomized) and small (3 μm). In addition, the geometry of the testing tool (entrance angle of a capillary die) might significantly affect determination of wall slip, and thus should be accounted for especially in the case of small and regular particles.

## Supplementary Material

Supplemental data
