# Supplementary material for: Effect of Particle Size and Shape on Wall Slip of Highly Filled Powder Feedstocks for Material Extrusion and Powder Injection Molding
Source: 3D Print Addit Manuf. 2023 Apr 12;10(2):236–44. doi: 10.1089/3dp.2021.0157 (PMC10122254; doi:10.1089/3dp.2021.0157)
Supplement: Supplemental data [file Supp_Fig1-2.docx]

Supplemental files

**Effect of Powder Particle Size and Shape on Wall Slip Velocity of Highly Filled Powder Injection Molding Feedstocks**

Daniel Sanetrnik, Berenika Hausnerova*, Martin Novak, Bhimasena Nagaraj Mukund

Dr. D. Sanetrnik

Centre of Polymer Systems, University Institute, Tomas Bata University in Zlin,

Trida T. Bati 5678, 760 01 Zlin, Czech Republic

E-mail: dsanetrnik@utb.cz, Telephone: +420576031726

Prof. B. Hausnerova – *corresponding author

Department of Production Engineering, Faculty of Technology, Tomas Bata University in Zlin, nam. T.G. Masaryka 5555, 760 01 Zlin, Czech Republic

Centre of Polymer Systems, University Institute, Tomas Bata University in Zlin,

Trida T. Bati 5678, 760 01 Zlin, Czech Republic

E-mail: hausnerova@utb.cz, Telephone: +420576035166

M. Novak

Department of Production Engineering, Faculty of Technology, Tomas Bata University in Zlin, nam. T.G. Masaryka 5555, 760 01 Zlin, Czech Republic

Centre of Polymer Systems, University Institute, Tomas Bata University in Zlin,

Trida T. Bati 5678, 760 01 Zlin, Czech Republic

E-mail: m8_novak@utb.cz

B. N. Mukund

Department of Production Engineering, Faculty of Technology, Tomas Bata University in Zlin, nam. T.G. Masaryka 5555, 760 01 Zlin, Czech Republic

Indo MIM Pvt. Ltd., KIADB Industrial Area, Hoskote, Bangalore 562114, India

E-mail: rao_nagaraj@utb.cz


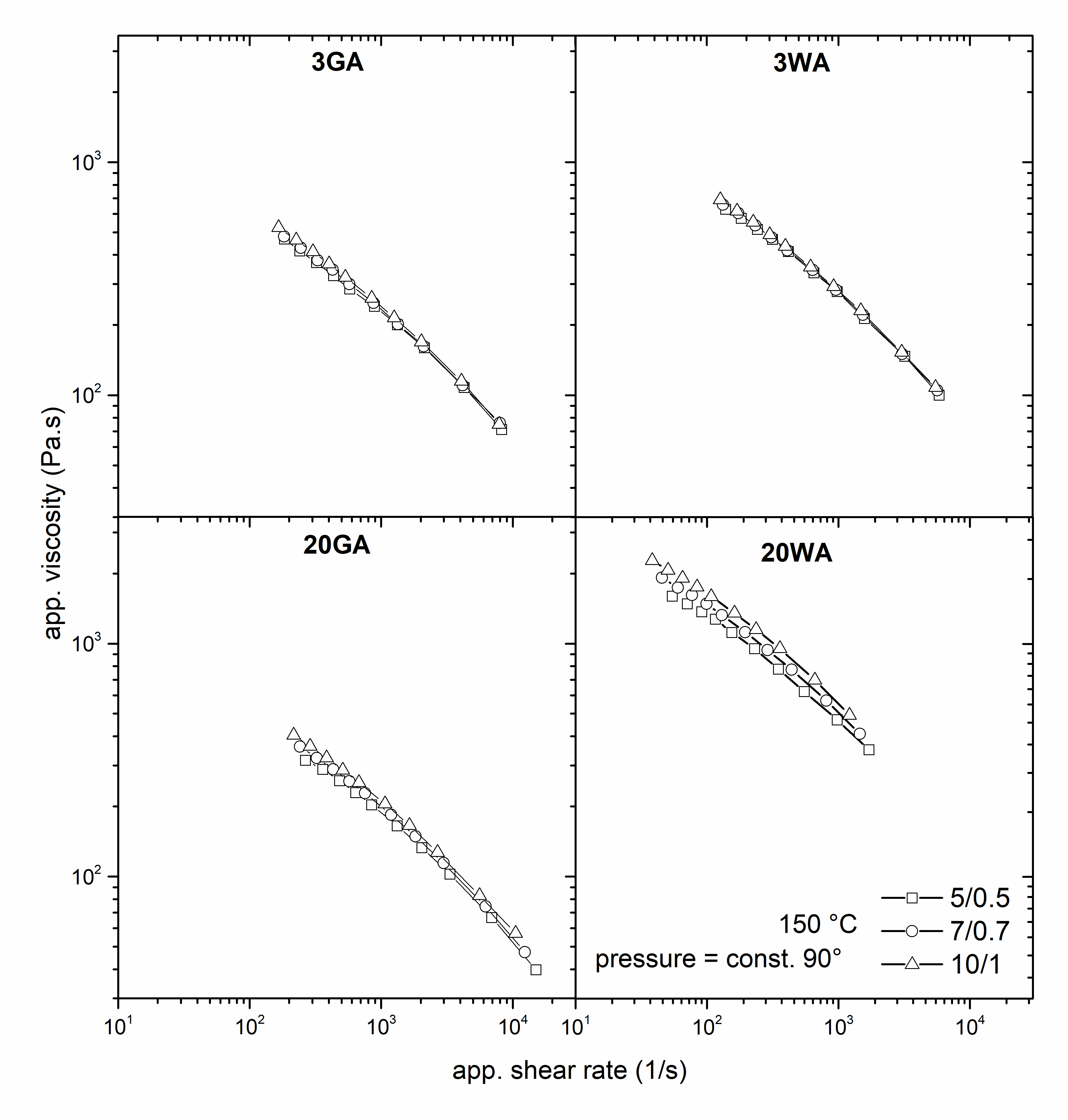
**Figure S1.** Apparent viscosity as a function of apparent shear rate of 316L feedstocks varying in size and shape obtained with conical capillary dies (entrance angle 90°)


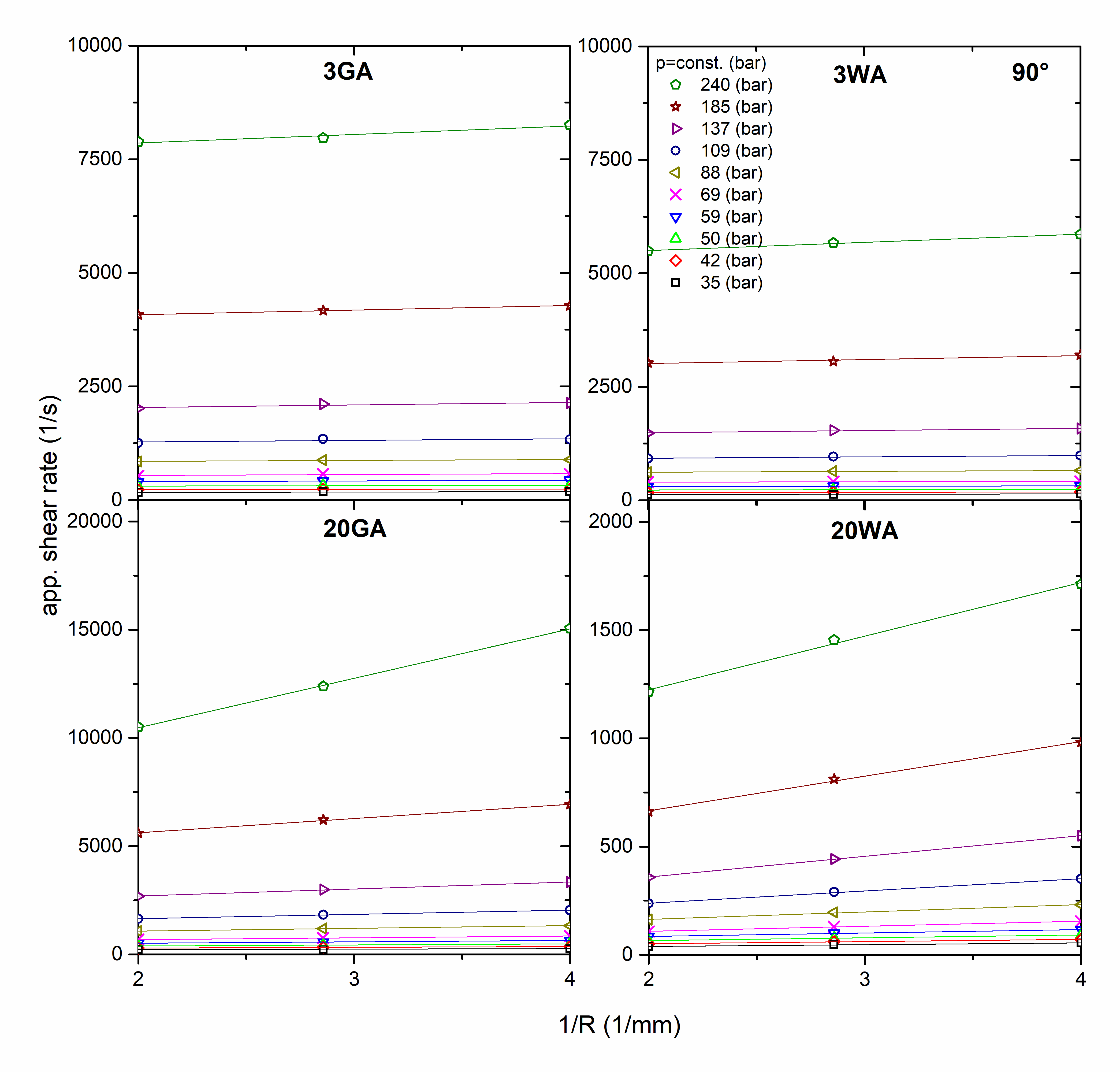


**Figure S2**. Mooney diagrams of 316L feedstocks varying in size and shape obtained with conical capillary dies (entrance angle 90°)
